# Detection of QTLs for panicle-related traits using an *indica* × *japonica* recombinant inbred line population in rice

**DOI:** 10.7717/peerj.12504

**Published:** 2021-11-29

**Authors:** Guan Li, Yichen Cheng, Man Yin, Jinyu Yang, Jiezheng Ying, Changlan Zhu

**Affiliations:** 1 School of Agricultural Sciences, Jiangxi Agricultural University, Nanchang, Jiangxi Province, China; 2 China National Rice Research Institute, Hangzhou, Zhejiang Province, China

**Keywords:** Rice, Panicle, Grain, Yield, Quantitative trait locus, Recombinant inbred line

## Abstract

**Background:**

The panicle is the most important organ in rice, and all the panicle-related traits are correlated with rice grain yield. Understanding the underlying genetic mechanisms controlling panicle development is very important for improving rice production.

**Methods:**

Nine panicle-related traits including heading date, panicle length, number of primary branches, number of secondary branches, number of grains per panicle, number of panicles per plant, number of filled grains per plant, seed-setting rate, and grain yield per plant were investigated. To map the quantitative trait loci (QTLs) for the nine panicle-related traits, a PCR-based genetic map with 208 markers (including 121 simple sequence repeats and 87 InDels) and a high-density linkage map with 18,194 single nucleotide polymorphism (SNP) markers were both used.

**Results:**

Using a recombinant inbred line population derived from an *indica* variety Huanghuazhan and a *japonica* line Jizi 1560, a total of 110 and 112 QTLs were detected for panicle-related traits by PCR-based genetic map and by high-density linkage map, respectively. Most of the QTLs were clustered on chromosomes 1, 2, 3, 6, and 7 while no QTLs were detected on chromosome 10. Almost all the QTLs with LOD values of more than 5.0 were repeatedly detected, indicating the accuracy of the two methods and the stability of the QTL effects. No genes for panicle-related traits have been previously reported in most of these regions. QTLs found in JD1006–JD1007 and RM1148–RM5556 with high LOD and additive values deserved further research. The results of this study are beneficial for marker-assisted breeding and provide research foundation for further fine-mapping and cloning of these QTLs for panicle-related traits.

## Introduction

Rice is one of the most important cereal crops about caloric intake and human nutrition (Yuan, 2014; Bhat et al., 2019). To meet the food requirements of the global population, an increase of agricultural production by more than 50% will be required by the year 2050 (Mumtaz et al., 2020), particularly in rice production in the developing countries in Asia and Africa. Therefore, improving rice yield has been and will always be the hot spot and frontier field in agricultural science. Panicle number and grain number per panicle are two of the three yield components (Fan et al., 2006; Niu et al., 2020). Generally, elite rice varieties with high yields will display better panicle architecture, including longer panicles, more primary branches, and more secondary branches (Ando et al., 2008). Panicle length (PL), which strongly impacts grain yield by affecting several panicle-related traits, can be used as a selection parameter for high-yield breeding (Wang et al., 2019b). The number of the primary branches (NPB) and the number of the secondary branches (NSB) are indispensable reference indicators in the breeding progress (Liang et al., 2019), and can influence the number of grains per panicle (NGPP), affecting the grain yield per plant (GYPP). However, the grain-filling traits including the number of filled grains per plant (NFGPP) and seed-setting rate (SSR) will directly affect GYPP (Ashikari et al., 2005; Niu et al., 2020). The transition from the vegetative stage to the reproductive stage is very crucial in plant development (Mo et al., 2020). Heading date (HD) significantly influences the grain yield and reflects the transition to reproductive growth, determining the regional adaptation of the variety (Endo-Higashi & Izawa, 2011). Therefore, these panicle-related traits are directly or indirectly correlated with rice yield, and correlations among these traits are worthy of further research and utilization in rice breeding.

With the rapid development of DNA molecular markers, quantitative trait locus (QTL) mapping has become a routine strategy to discover genes involved in complex quantitative traits (Kebriyaee et al., 2012; Wang et al., 2019c). Phenotypic examination and genotypic identification of the mapping populations are two essential components for QTL analysis (Wu et al., 2020). Even if all the rice materials are planted in the same field and cultivated under uniform conditions, noise from environmental effects cannot be completely removed, and pleiotropic effects of genes for non-target traits still exist, which both can affect the phenotype (Ando et al., 2008).

Traditionally, genotyping of the mapping population is conducted using hundreds of PCR-based DNA markers such as simple sequence repeats and InDels. With the development of next-generation sequencing, a genotyping-by-resequencing method such as specific length amplified fragment sequencing (SLAF-seq) with high resolution has been applied in linkage mapping (Wu et al., 2020). By using the high-density map, the confidence interval of the QTL detected can be narrowed.

To date, more than 900 QTLs for grain number per plant have been identified in the Gramene database (http://www.gramene.org/). Only some of these QTLs have been fine-mapped and cloned. *Gn1a*, the first QTL for grain number per plant, was cloned on the short arm of chromosome 1 using the Habataki/Koshihikari near-isogenic line (NIL) population (Ashikari et al., 2005), which can also increase the grain yield. *Ghd7*, which is fine-mapped on chromosome 7 using recombinant inbred line (RIL) population from Zhenshan 97/Minghui 63 (Xue et al., 2008), controls NGPP, HD, and plant height simultaneously. However, there are still many QTLs for the panicle-related traits to be detected and fine-mapped. The underlying genetic and molecular mechanisms regulating panicle-related traits remain largely unknown. Molecular characterization of genes controlling panicle-related traits will not only be valuable for high-yield breeding in rice but also be beneficial for the exploration of the regulatory mechanisms and identification of the regulatory networks among these traits.

The objective of this study was to analyze the relationships between several panicle-related traits and to detect QTLs regulating panicle development, panicle structure, and grain filling by two mapping methods using RIL derived from a cross between an *indica* rice variety Huanghuazhan (HHZ) and a *japonica* rice accession Jizi 1560 (JZ1560). QTLs detected with both methods in both experimental years with higher LOD, additive effect, and proportion of the phenotypic variance explained were selected and regarded as definitive QTLs. The findings detailed herein will contribute to enhancing the yield of rice.

## Materials & methods

### Plant materials and field trials

The RIL population derived from an *indica* rice variety HHZ and a *japonica* rice accession JZ1560, consisting of 280 RILs, was constructed using the single-seed descent method by Ying et al. (2018). The RIL population was planted for two continuous years (2015 and 2016) in a randomized complete block design, in the paddy fields of the China National Rice Research Institute, Hangzhou, China, with the spacing of 16.6 cm × 26.7 cm during the rice-growing seasons. Eighteen plants for each RIL were transplanted and the middle four plants were harvested after maturity. Field management was conducted following the local standard practice in rice production.

### Phenotypic evaluation

The date when the first panicle stretched out of the flag leaf sheath was recorded for each plant, and the average duration from the seeding date to the recorded date of the eighteen plants was calculated as the HD for the line. The number of panicles per plant (NPPP), NPB, NSB, and PL were measured before threshing. After threshing, the number of unfilled grains was counted. The GYPP and NFGPP were measured by the method of Zhang et al. (2016). Then, the NGPP and SSR were calculated.

### DNA marker analysis and genetic map

DNA was extracted using the young leaves following the method of Zheng et al. (1995). PCR was performed in a 10-µL reaction system containing 2 µL of DNA template (approximately 50 ng), 0.1 µL of 100 mol/L each primer, 5 µL of 2 × *Taq* MasterMix (Shangya Biotech, Hangzhou, China), and 2.8 µL of ddH_2_O. PCR program was performed with an initial denaturation at 94 °C for 2 min, 30 cycles of denaturation at 94 °C for 30 s, annealing at 55 °C for 30 s, and extension at 72 °C for 30 s, and a final extension at 72 °C for 2 min. The PCR products were visualized on 2.5% agarose gels using GelRed staining (Biotium, Fremont, CA, USA). A total of 208 markers, including 121 simple sequence repeats and 87 InDel markers were used to construct the low-density PCR-based linkage map (Wu et al., 2020). A total of 18,194 single nucleotide polymorphism (SNP) markers spanned all the 12 rice chromosomes with an average genetic distance of 0.12 cM were used to construct the high-density linkage map (Ying et al., 2018; Wu et al., 2020).

### QTL mapping

Permutations for each trait of the population were performed. The test with 1,000 permutations at the 0.01 probability level suggested a LOD threshold of 2.5 to identify a putative QTL. For the low-density PCR-based genetic map, QTLs were identified with Windows QTL Cartographer 2.5. For the high-density genetic map, QTL analysis was performed with R/qtl software. QTL analysis was conducted with the composite interval mapping method and LOD 2.5 was used as the threshold for detecting a putative QTL.

### Data analysis

Descriptive statistics, including range, mean, standard deviation, skewness, and kurtosis, were calculated with Microsoft Excel 2013. The mean values of each trait were used to calculate the correlation coefficients. Correlation analysis was carried out by SAS software (Version 6.1).

## Results

### Phenotypic variance

**Figure 1:**
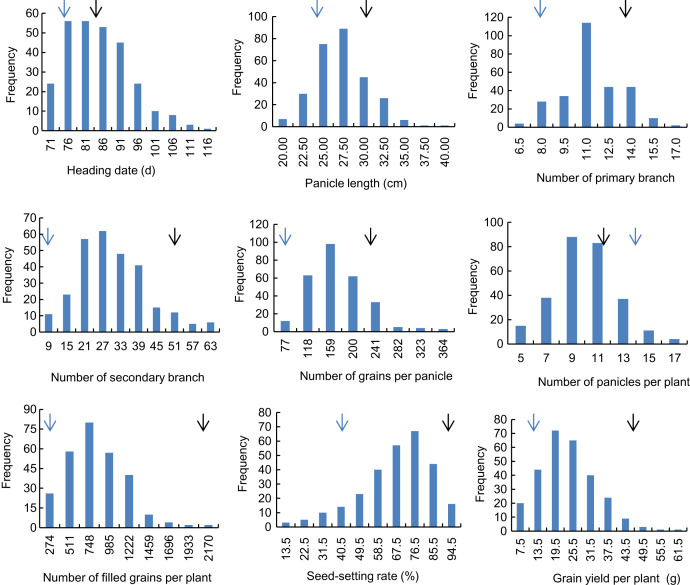
Frequency distribution of the nine panicle-related traits in 2015. Black arrows indicate the phenotype of Huanghuazhan and blue arrows indicate the phenotype of Jizi 1560.

**Figure 2:**
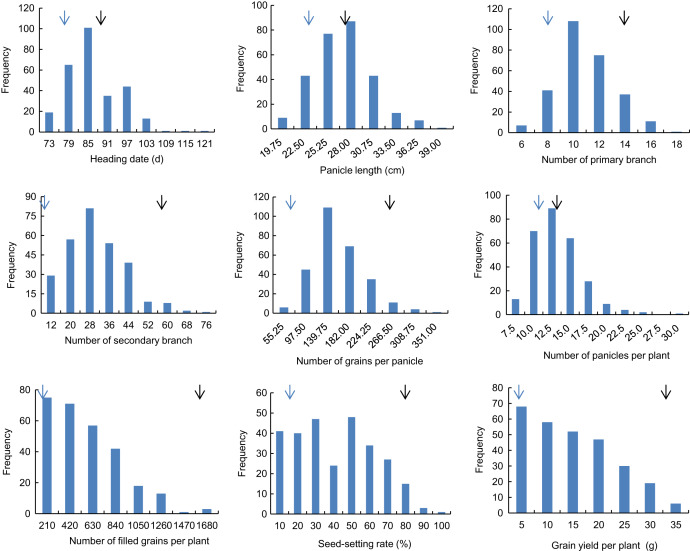
Frequency distribution of the nine panicle-related traits in 2016. Black arrows indicate the phenotype of Huanghuazhan and blue arrows indicate the phenotype of Jizi 1560.

Descriptive statistics of the panicle-related traits, including HD, PL, NPB, NSB, NPPP, NGPP, NFGPP, SSR and GYPP, are shown in Table 1. Except for NGPP and NPPP, all the other traits were continuously distributed with low kurtosis and low skewness, showing a typical pattern of quantitative variation. Significant differences were observed between the two parents for all the panicle-related traits. Frequency distributions of the nine panicle-related traits in the two years are shown in Figs. 1 and 2. Significant transgressive and continuous segregations were observed in almost all the nine traits, which indicated that these traits were controlled by many genes.

**Table 1:** Statistical data of the nine panicle-related traits in 2015 and 2016.

Year	Trait	RIL							HHZ	JZ
		Min	Max	Mean	SD	CV	Skew	Kurt		
2015	HD	68	115	82.9	9.1	0.1	0.687	0.158	82.0	73.0
	PL	17.6	39.8	26.0	3.3	0.1	0.460	0.915	29.1	23.4
	NPB	5	16	10.9	2.0	0.2	0.024	−0.007	13.0	7.0
	NSB	4	61	27.5	11.6	0.4	0.547	0.234	50.0	5.0
	NGPP	38	362	152.2	51.7	0.3	0.983	2.083	228.0	52.0
	NPPP	3.3	18.3	9.2	2.6	0.3	0.464	0.810	10.8	13.0
	NFGPP	37.5	2169.5	710.2	351.1	0.5	0.789	1.247	2169.5	207.3
	SSR	4.8	94.0	62.6	17.4	0.3	−0.829	0.465	88.3	36.3
	GYPP	1.8	61.2	20.8	9.7	0.5	0.639	0.806	44.8	9.8
2016	HD	68	120	83.9	8.5	0.1	0.595	0.720	85.0	75.0
	PL	17.5	38.9	25.7	3.5	0.1	0.404	0.380	26.7	21.9
	NPB	6	17	10.5	2.1	0.2	0.402	−0.001	13.0	7.0
	NSB	4	75	27.3	12.2	0.4	0.680	0.634	54.0	4.0
	NGPP	14	350	138.7	49.8	0.4	0.782	1.181	252.0	53.0
	NPPP	5.7	29.8	12.0	3.4	0.3	1.239	3.174	11.8	9.8
	NFGPP	0.4	1645.0	456.7	329.7	0.7	0.822	0.511	1584.5	56.3
	SSR	0.0	97.9	35.8	22.0	0.6	0.240	−0.838	75.1	12.7
	GYPP	0.0	34.1	12.2	8.2	0.7	0.452	−0.664	30.5	2.4

**Note:**

RIL, Recombinant inbred line; HHZ, Huanghuazhan; JZ, Jizi 1560; HD, Heading date; PL, Panicle length; NPB, Number of primary branches; NSB, Number of secondary branches; NGPP, Number of grains per panicle; NPPP, Number of panicles per plant; NFGPP, Number of filled grains per panicle; SSR, Seed-setting rate; GYPP, Grain yield per plant.

### Correlation analysis of the nine panicle-related traits

**Figure 3:**
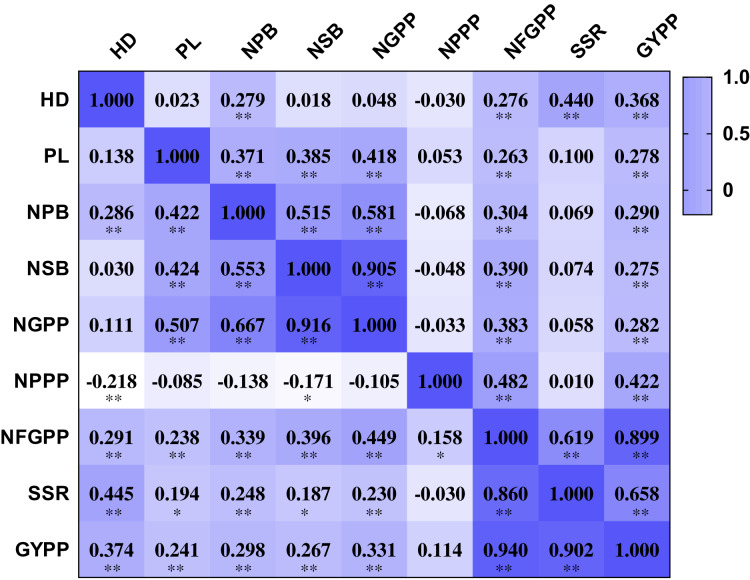
Correlation among the nine panicle-related traits in 2015 and 2016. HD, Heading date; PL, Panicle length; NPB, Number of primary branches; NSB, Number of secondary branches; NGPP, Number of grains per panicle; NPPP, Number of panicles per plant; NFGPP, Number of filled grains per panicle; SSR, Seed-setting rate; GYPP, Grain yield per plant. Values shown in the upper part are data for 2015 and values shown in the bottom part are data for 2016. Asterisks (* and **) indicate significant correlations at the 0.01 and 0.001 levels, respectively.

The nine panicle-related traits were closely related to each other. The NPPP showed negative correlations with several panicle-related traits, including HD, NPB, NSB and NGPP. However, most of the correlation coefficients were not significant at the 0.01 and 0.001 levels. The other eight traits were all positively related to each other. The HD showed positive correlation coefficients with NPB, NFGPP, SSR, and GYPP in both two years. The correlation coefficients ranged from 0.276 to 0.445 and were all significant at the 0.001 level (Fig. 3). The PL showed positive correlation coefficients with NPB, NSB, NGPP, NFGPP, and GYPP in both years, and the correlation coefficients ranged from 0.238 to 0.507, which were all significant at the 0.001 level. The NPB was closely related to NSB, NGPP, NFGPP, SSR, and GYPP. NSB was highly related to NGPP and the correlation coefficients were 0.905 in 2015 and 0.916 in 2016. The NFGPP, SSR, and GYPP were all closely related to each other with coefficients higher than 0.619. However, most of the coefficients between NPPP and the other panicle-related traits were not significant.

### QTLs detected by PCR-based low-density genetic map

**Figure 4:**
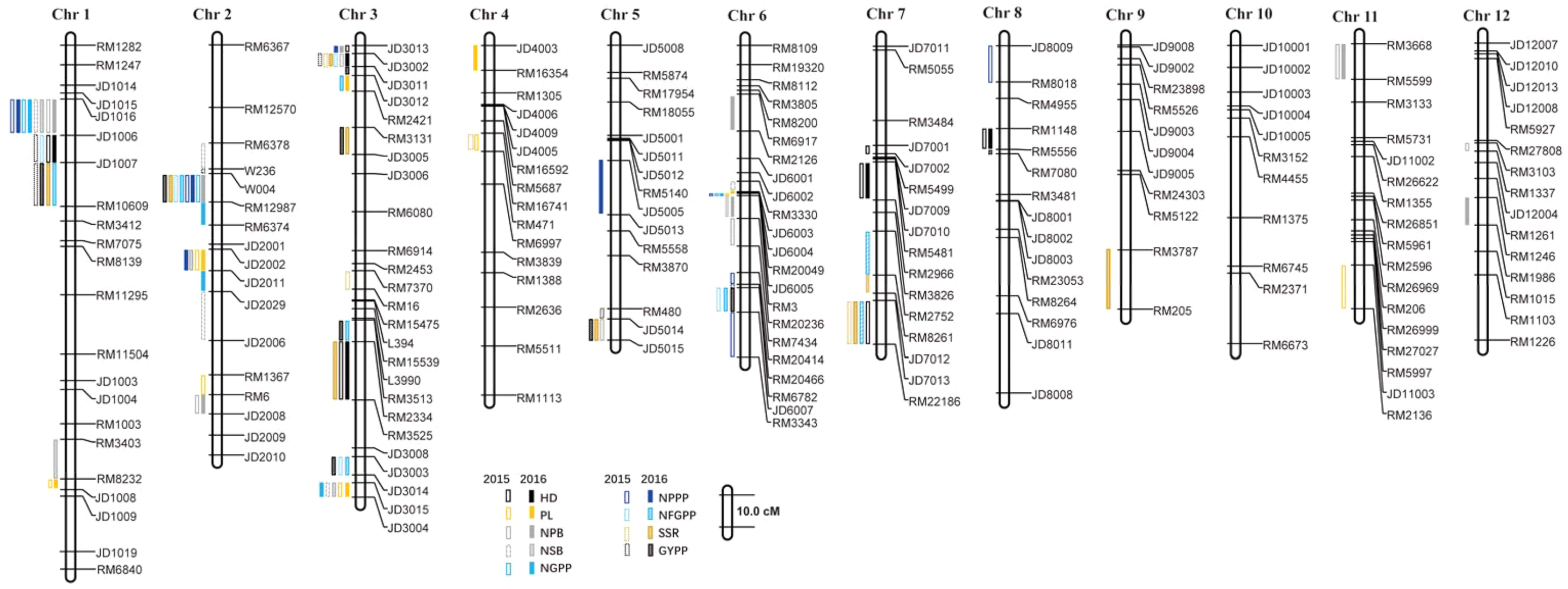
QTLs for panicle-related traits by PCR-based linkage map in 2015 and 2016. RIL, Recombinant inbred line; HHZ, Huanghuazhan; JZ, Jizi 1560; HD, Heading date; PL, Panicle length; NPB, Number of primary branches; NSB, Number of secondary branches; NGPP, Number of grains per panicle; NPPP, Number of panicles per plant; NFGPP, Number of filled grains per panicle; SSR, Seed-setting rate; GYPP, Grain yield per plant.

A total of 110 QTLs were detected by the PCR-based low-density genetic map in the HHZ/JZ1560 RIL population in the two experimental years. These QTLs were distributed on almost all the 12 chromosomes except chromosome 10 (Fig. 4). The highest number of QTLs was located on chromosome 3 (28 QTLs), followed by chromosome 2 (20 QTLs), chromosome 1 (19 QTLs), and chromosome 6 (16 QTLs).

In 2015, 51 QTLs were detected for the nine panicle-related traits, including seven for HD, seven for PL, seven for NPB, five for NSB, three for NGPP, six for NPPP, five for NFGPP, four for SSR, and seven for GYPP (Fig. 4; Table 2). In 2016, a total of 59 QTLs were detected, including five for HD, six for PL, eight for NPB, five for NSB, five for NGPP, five for NPPP, eight for NFGPP, nine for SSR and eight for GYPP (Fig. 4; Table 3). Among these QTLs, 30 were located at the same or adjacent regions.

**Table 2:** QTLs detected by PCR-based low-density mapping in 2015.

Trait	Chr	Marker interval	Position	LOD	*A*	*R* ^2^
HD	1	JD1006–JD1007	0.4111	5.951	−2.209	0.614
HD	3	JD3013–JD3002	0.0201	22.903	−3.997	0.617
HD	3	RM2334–RM3525	1.1331	3.993	−1.746	0.640
HD	7	JD7001–JD7002	0.3121	3.176	−1.363	0.601
HD	7	RM5481–RM2966	0.3641	3.953	−1.551	0.606
HD	7	JD7013–RM22186	0.9301	5.481	−1.827	0.608
HD	8	RM1148–RM5556	0.3011	35.788	5.509	0.653
PL	1	RM8232–JD1008	1.5911	4.930	0.831	0.304
PL	2	JD2002–JD2011	0.7391	5.874	−0.901	0.303
PL	2	RM1367–RM6	1.2061	5.662	0.952	0.319
PL	3	JD3015–JD3004	1.6321	5.331	−0.856	0.303
PL	4	RM16741–RM471	0.3671	3.265	0.702	0.309
PL	6	JD6005–RM3	0.5361	2.569	−0.586	0.331
PL	11	JD11003–RM2136	0.8171	3.154	−0.669	0.342
NPB	1	JD1016–JD1006	0.2511	5.365	−0.624	0.320
NPB	2	RM6–JD2008	1.2581	3.008	0.406	0.291
NPB	3	JD3002–JD3011	0.0291	4.814	−0.483	0.285
NPB	5	JD5014–JD5015	1.0311	2.566	0.379	0.321
NPB	6	RM20236–RM7434	0.6771	2.661	−0.382	0.321
NPB	11	RM3668–RM5599	0.0501	3.767	−0.485	0.301
NPB	12	RM3103–RM1337	0.3471	3.084	−0.431	0.289
NSB	1	JD1016–JD1006	0.3011	11.236	−4.993	0.326
NSB	2	RM6378–W236	0.3551	3.028	−2.313	0.294
NSB	2	JD2029–JD2006	0.9061	3.254	−2.523	0.300
NSB	3	JD3015–JD3004	1.6321	2.840	−2.165	0.292
NSB	6	RM3330–JD6003	0.5121	4.699	−2.974	0.294
NGPP	1	JD1016–JD1006	0.2911	8.118	−19.202	0.289
NGPP	2	W004–RM12987	0.4761	4.743	−13.656	0.271
NGPP	6	JD6005–RM3	0.5361	4.630	−13.010	0.261
NPPP	1	JD1016–JD1006	0.2111	3.427	0.605	0.296
NPPP	2	W004–RM12987	0.4861	8.894	−0.997	0.320
NPPP	6	JD6005–RM3	0.5361	4.056	0.656	0.290
NPPP	6	RM20414–RM20466	0.8641	5.723	−0.773	0.292
NPPP	6	JD6007–RM3343	1.0401	2.710	−0.590	0.264
NPPP	8	JD8009–RM8018	0.1101	3.643	0.589	0.290
NFGPP	1	JD1006–JD1007	0.3411	5.280	−92.898	0.353
NFGPP	2	W004–RM12987	0.4661	15.207	−153.345	0.351
NFGPP	3	JD3002–JD3011	0.0391	4.996	−89.167	0.357
NFGPP	3	JD3003–JD3014	1.5391	2.946	−67.186	0.353
NFGPP	6	RM6782–JD6007	0.9111	3.140	−71.963	0.353
SSR	3	JD3002–JD3011	0.0291	10.102	−6.434	0.275
SSR	3	RM7370–RM16	0.8151	3.819	−4.101	0.275
SSR	4	RM16741–RM471	0.3371	4.559	4.574	0.289
SSR	7	JD7013–RM22186	0.9301	2.709	−3.373	0.277
GYPP	1	JD1006–JD1007	0.3711	3.073	−2.310	0.287
GYPP	1	JD1007–RM10609	0.5021	2.570	−2.298	0.288
GYPP	2	W236–W004	0.4591	4.587	−2.451	0.242
GYPP	3	JD3002–JD3011	0.0391	6.205	−3.001	0.249
GYPP	5	RM480–JD5014	0.9541	2.809	−1.900	0.240
GYPP	6	RM20466–RM6782	0.8691	4.371	−2.386	0.237
GYPP	8	RM5556–RM7080	0.3261	2.945	1.994	0.240

**Note:**

HD, Heading date; PL, Panicle length; NPB, Number of primary branches; NSB, Number of secondary branches; NGPP, Number of grains per panicle; NPPP, Number of panicles per plant; NFGPP, Number of filled grains per panicle; SSR, Seed-setting rate; GYPP, Grain yield per plant; *A*, Additive effect of replacing a Jizi 1560 allele with a Huanghuazhan allele; *R*^2^, Proportion of the phenotypic variation explained by the QTL.

**Table 3:** QTLs detected by PCR-based low-density mapping in 2016.

Trait	Chr	Marker	Position	LOD	*A*	*R* ^2^
HD	1	JD1006–JD1007	0.3711	7.222	−2.488	0.556
HD	3	JD3002–JD3011	0.0291	13.976	−3.013	0.529
HD	3	RM2334–RM3525	1.1831	3.871	−1.866	0.549
HD	7	RM5481–RM2966	0.3641	2.869	−1.310	0.551
HD	8	RM1148–RM5556	0.3011	33.063	5.367	0.603
PL	1	RM8232–JD1008	1.5911	2.920	0.665	0.297
PL	2	JD2002–JD2011	0.7391	7.750	−1.118	0.262
PL	3	JD3012–RM2421	0.1091	3.980	−0.788	0.261
PL	3	JD3015–JD3004	1.6321	3.759	−0.775	0.264
PL	4	JD4003–RM16354	0.0301	3.035	0.743	0.271
PL	6	JD6003–JD6004	0.5301	4.378	−0.851	0.259
NPB	1	JD1016–JD1006	0.3111	3.164	−0.474	0.287
NPB	2	W004–RM12987	0.5161	8.577	−0.780	0.317
NPB	2	RM6–JD2008	1.2481	3.179	0.426	0.322
NPB	3	JD3013–JD3002	0.0101	3.358	−0.447	0.285
NPB	6	RM6917–RM2126	0.2871	3.693	−0.526	0.334
NPB	6	RM3–RM20236	0.6041	3.661	−0.497	0.334
NPB	11	RM3668–RM5599	0.0601	5.214	−0.638	0.319
NPB	12	RM1246–RM1986	0.5061	2.845	−0.425	0.283
NSB	1	JD1016–JD1006	0.3111	15.504	−5.505	0.458
NSB	1	RM3403–RM8232	1.4471	2.825	−2.047	0.435
NSB	2	JD2002–JD2011	0.7791	10.881	−4.373	0.456
NSB	3	JD3015–JD3004	1.6321	7.143	−3.285	0.435
NSB	6	RM3–RM20236	0.5741	8.731	−3.989	0.452
NGPP	1	JD1016–JD1006	0.3211	15.445	−22.546	0.429
NGPP	2	RM12987–RM6374	0.5791	3.055	−12.334	0.398
NGPP	2	JD2011–JD2029	0.8471	5.206	−13.536	0.426
NGPP	3	JD3015–JD3004	1.6321	5.804	−12.295	0.412
NGPP	6	JD6005–RM3	0.5361	6.526	−13.375	0.411
NPPP	1	JD1016–JD1006	0.2811	3.994	0.946	0.270
NPPP	2	W004–RM12987	0.4661	9.092	−1.433	0.244
NPPP	2	JD2002–JD2011	0.7391	2.942	0.786	0.240
NPPP	3	JD3013–JD3002	0.0001	3.294	0.703	0.240
NPPP	5	JD5005–JD5013	0.5171	2.926	0.817	0.268
NFGPP	1	JD1007–RM10609	0.4921	9.345	−131.620	0.439
NFGPP	2	W004–RM12987	0.4861	11.396	−124.556	0.399
NFGPP	3	JD3012–RM2421	0.1191	5.308	−82.404	0.388
NFGPP	3	RM3513–RM2334	1.0641	2.613	−58.430	0.410
NFGPP	3	JD3003–JD3014	1.5391	5.238	−84.026	0.393
NFGPP	6	RM6782–JD6007	0.9011	3.139	−62.747	0.418
NFGPP	7	RM2752–RM8261	0.6821	4.216	−79.212	0.397
NFGPP	7	JD7013–RM22186	0.8901	3.223	−71.042	0.386
SSR	1	JD1007–RM10609	0.5021	8.392	−8.951	0.413
SSR	2	W004–RM12987	0.5161	5.904	−6.351	0.360
SSR	3	JD3002–JD3011	0.0291	7.570	−6.600	0.336
SSR	3	RM3131–JD3005	0.3361	2.535	−5.809	0.378
SSR	3	RM2334–RM3525	1.1031	6.219	−6.813	0.356
SSR	5	JD5014–JD5015	1.0111	3.516	−4.684	0.343
SSR	7	RM8261–JD7012	0.7311	2.587	−3.904	0.366
SSR	7	JD7013–RM22186	0.8901	3.158	−4.731	0.382
SSR	9	RM3787–RM205	0.7541	3.286	4.894	0.386
GYPP	1	JD1007–RM10609	0.4921	7.389	−3.265	0.364
GYPP	2	W004–RM12987	0.5061	5.604	−2.355	0.318
GYPP	3	JD3011–JD3012	0.0961	5.931	−2.359	0.310
GYPP	3	RM3131–JD3005	0.3161	2.893	−2.072	0.339
GYPP	3	RM3513–RM2334	1.0641	4.102	−1.980	0.303
GYPP	3	JD3003–JD3014	1.5491	2.644	−1.499	0.331
GYPP	5	JD5014–JD5015	0.9911	3.198	−1.642	0.300
GYPP	6	RM6782–JD6007	0.8911	2.579	−1.500	0.331

**Note:**

HD, Heading date; PL, Panicle length; NPB, Number of primary branches; NSB, Number of secondary branches; NGPP, Number of grains per panicle; NPPP, Number of panicles per plant; NFGPP, Number of filled grains per panicle; SSR, Seed-setting rate; GYPP, Grain yield per plant; *A*, Additive effect of replacing a Jizi 1560 allele with a Huanghuazhan allele; *R*^2^, Proportion of the phenotypic variation explained by the QTL.

There were several QTL clusters (with more than five QTLs) on chromosomes 1, 2, 3, and 6, located in the regions of JD1016–RM10609 on chromosome 1, RM6378–RM6374 and JD2002–JD2006 on chromosome 2, JD3013–RM2421 and RM3513–RM3525 on chromosome 3, as well as RM3330–RM7437 and RM20414–RM3343 on chromosome 6 (Fig. 4).

### QTLs detected by high-density SLAF linkage map

**Figure 5:**
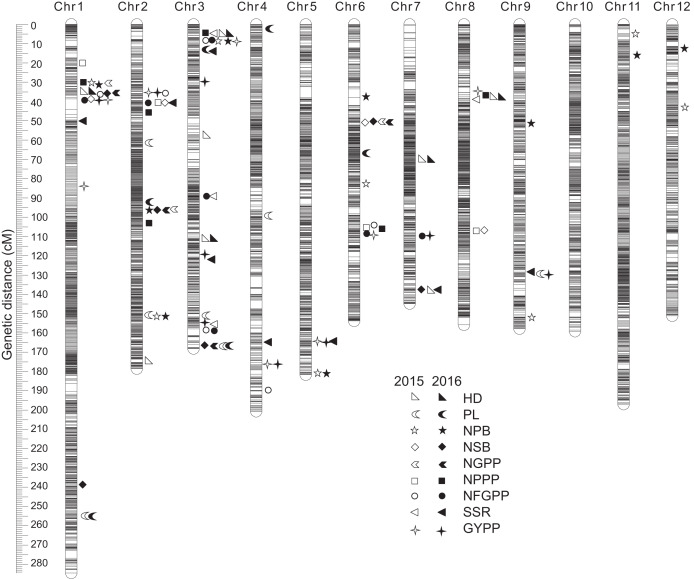
QTLs for panicle-related traits by high-density linkage map in 2015 and 2016. HD, Heading date; PL, Panicle length; NPB, Number of primary branches; NSB, Number of secondary branches; NGPP, Number of grains per panicle; NPPP, Number of panicles per plant; NFGPP, Number of filled grains per panicle; SSR, Seed-setting rate; GYPP, Grain yield per plant.

A total of 112 QTLs were detected by the high-density genetic map in the RIL population in the two years. These QTLs were also distributed on almost all the 12 chromosomes except chromosome 10 (Fig. 5). The highest number of QTLs were also located on chromosome 3 (28 QTLs), followed by chromosome 2 (19 QTLs), chromosome 1 (19 QTLs), and chromosome 6 (12 QTLs).

**Figure 6:**
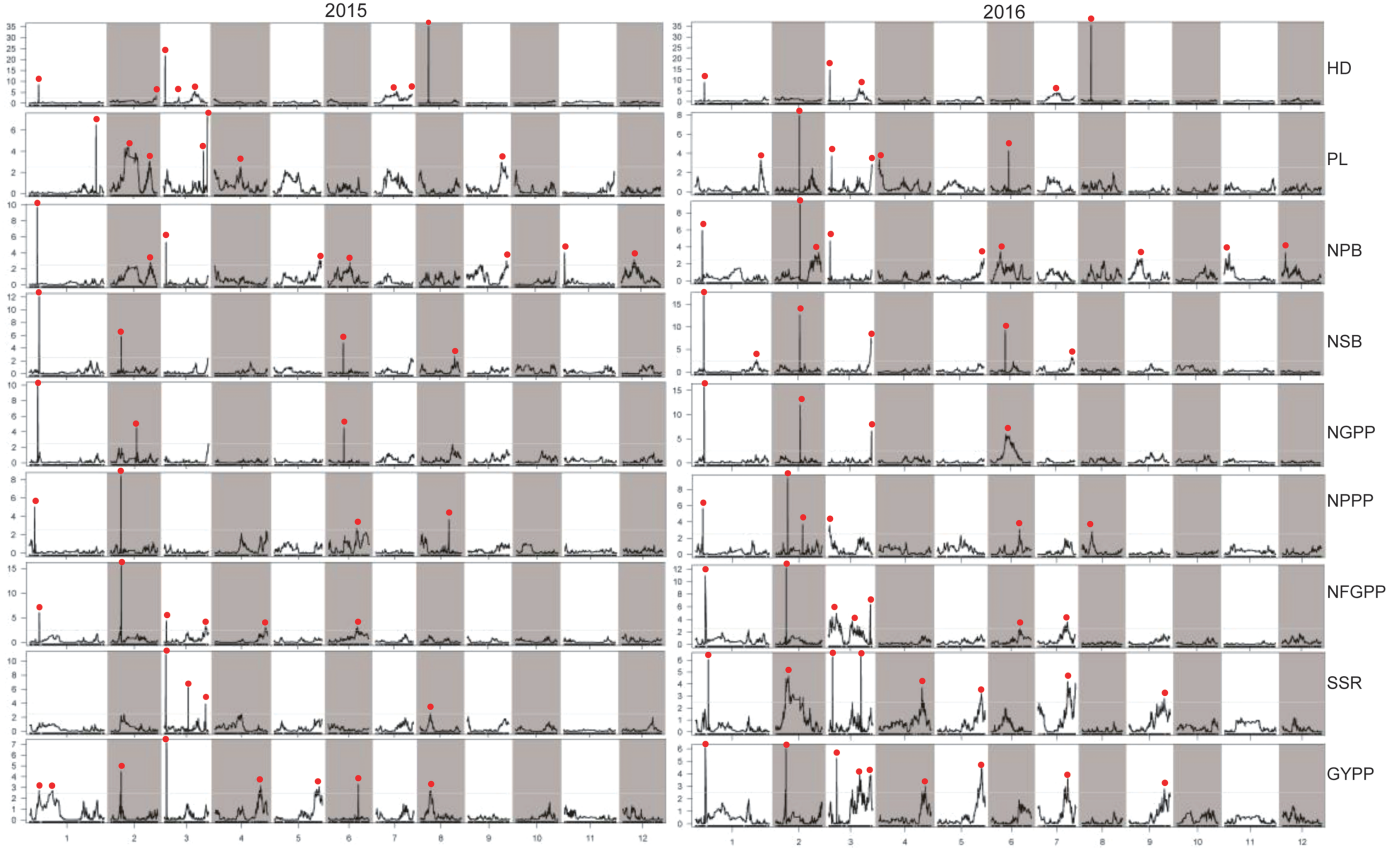
LOD values of the QTLs for panicle-related traits detected by high-density linkage map in both the two years. HD, Heading date; PL, Panicle length; NPB, Number of primary branches; NSB, Number of secondary branches; NGPP, Number of grains per panicle; NPPP, Number of panicles per plant; NFGPP, Number of filled grains per panicle; SSR, Seed-setting rate; GYPP, Grain yield per plant.

In 2015, 52 QTLs were detected for the nine panicle-related traits, including eight for HD, seven for PL, eight for NPB, four for NSB, three for NGPP, four for NPPP, six for NFGPP, four for SSR, and eight for GYPP (Fig. 6; Table S1). In 2016, a total of 60 QTLs were detected, including five for HD, six for PL, nine for NPB, six for NSB, four for NGPP, six for NPPP, seven for NFGPP, eight for SSR, and nine for GYPP (Fig. 6; Table S2). Among these QTLs, 29 were located at the same or adjacent regions.

### Stable QTLs detected by the two maps across the two years

Considering the effect of colocalization, we further analyzed the regions with the QTLs detected in this study. Taken together, a total of 93 regions with QTLs for panicle-related traits detected were found, and 64 regions with QTLs located in the same or adjacent loci were found at least twice. However, 27 regions with QTLs detected by both the two maps in both years were found (Tables 2, 3, S1 and S2). Among the QTLs with a LOD value of more than 5.0, only one QTL for NPPP was detected once, which indicated that QTLs with high LOD values were stable and could be detected in different environments or by different mapping methods. Among the 93 regions, only 19 contained cloned genes for panicle-related traits. Since no genes for panicle-related traits have been previously reported in the regions of JD1006–JD1007 and RM1148–RM5556, QTLs found in these regions with high LOD and additive values deserved further research. To further fine-map and clone the QTLs for panicle-related traits, recombinant plants with sequential heterozygous regions should be used to narrow down the target regions.

## Discussion

Quantitative traits are generally controlled by several major genes and many minor genes simultaneously. Although many genes for the HD, grain size and grain yield-related traits have been previously cloned, there are still many genes/QTLs distributed on all the 12 chromosomes waiting for exploitation. In the last two decades, QTL mapping is still a major approach to characterize the contribution of individual genomic regions (Zhu et al., 2016).

Several cloned genes related to panicle development have been previously reported on chromosome 1. *Gn1a/OsCKX2* and *SMALL GRAIN 11* (*SMG11*) have been cloned between JD1016 and JD1006 (Ashikari et al., 2005; Fang et al., 2019). *Gn1a*, encoding cytokinin dehydrogenase precursor, is a major QTL controlling grain number per panicle (Ashikari et al., 2005). *SMG11* encoding cytochrome P450 regulates grain size, grain number, and grain yield by catalyzing the synthesis of brassinosteroids (BRs) (Fang et al., 2019). A gene regulating the synthesis of BRs, named *Ovate Family Protein 1* (*OFP1*), is located in the interval of JD1006 and JD1007, and its overexpression lines show lower plant height and panicle number (Xiao et al., 2017). Between RM3403 and RM8232, there is also a cloned gene related to panicle traits, *monoculm 2* (*MOC2*), encoding fructose-1,6-bisphosphatase, a key enzyme regulating the synthesis of sucrose, and the mutant *moc2* shows only one tiller, lower plant height, smaller panicle, and fewer grains per panicle (Koumoto et al., 2013).

On chromosome 2, *LARGE1*/*OML4* (Lyu et al., 2020) and *EP3/LP* (Piao et al., 2009; Li et al., 2011a) were detected in this study. The mutant *large1* exhibits higher plant height, larger grains, longer panicles, more primary branches, but lower grains per panicle and lower secondary branches (Lyu et al., 2020). OML4 can interact with a protein kinase GSK2, which is a crucial regulatory factor during the signal transduction of BRs and can interact with many proteins to regulate the growth and development of rice plants (Lyu et al., 2020). *SDG725* encoding an H3K36 methyltransferase plays a pivotal role in development of the rice plants by mediating the expression of BR-related genes. *LP* encodes an OsFBK5-F-box domain and Kelch repeat-containing protein, and the mutant produces more grains and branches, especially NPB, and higher GYPP (Li et al., 2011a). LP can interact with OsCKX2, a cytokinin oxidase, and regulate the concentration of cytokinin in rice tissues to improve the architecture of the rice plants and the grain yield. Two QTL clusters for the panicle-related traits were detected in the regions of the two genes on chromosome 2 in this study, demonstrating the effects of *LARGE1*/*OML4* and *EP3*/*LP* in the population. *OsGRF4*/*GS2*/*GL2* (Che et al., 2015; Duan et al., 2015; Hu et al., 2015) encodes a growth regulator, and the overexpression of *GS2* can significantly enhance the growth of cells and further improve grain weight and grain yield. GRF4, which is a crucial element in the signal transduction of gibberellin, can promote the uptake and assimilation of nitrogen, and can also accelerate the metabolism of carbohydrates, influencing the grain size, panicle length, and grain yield (Hu et al., 2015). However, QTLs for panicle-related traits have not been detected at the location of *OsGRF4* in this study. In the interval of RM6–JD2008, *DTH2*, *OsMADS57*, and *Ghd2* have been previously cloned. Wu et al. (2013) cloned *DTH2*, a minor QTL for HD, while Guo et al. (2013) have reported that overexpression of *OsMADS57* can significantly increase the tillering number and Liu et al. (2016) indicated that overexpression of *Ghd2* can increase the NGPP and plant height, and can also accelerate heading and leaf senescence. However, QTLs for the related traits were not detected, and only QTLs for the NPB were detected in the interval of RM6–JD2008 in this study. This may be due to the complex genetic background or due to the effects of environment and genetic interaction.

Three QTL clusters were identified on chromosome 3. *DTH3* (Bian et al., 2011)*/OsMADS50* (Lee et al., 2004), a MADS-box protein gene for HD, is located in one of the QTL clusters. Near the location of *DTH3*, QTLs for HD were detected in both years by both methods, indicating the stability of the QTL and the accuracy of the two mapping methods. In the interval of RM3513 and RM2334, QTLs for NFGPP and GYPP were detected in 2016. Qi et al. (2012) and Zhang et al. (2012a) also found a gene for grain size *GL3.1*/*OsPPKL1* in this region, which can also regulate grain yield. In the interval of RM2334 and RM3525, there is a heading date gene *OsPIPK1*, which can regulate flowering by several signal pathways such as abscisic acid, gibberellin, auxin, and cytokinin (Ma et al., 2004). The effect of *OsPIPK1* was also detected in this study. At the end of chromosome 3, Ying et al. (2018) have cloned a major QTL for grain size, named *TGW3*. Several QTLs for panicle-related traits were also detected in the region of *TGW3* in this study.

In the intervals of the QTLs on chromosome 6 detected in this study, five genes related with panicle traits including *OsJMT1* (Kim et al., 2009), *TGW6* (Ishimaru et al., 2013), *GW6a* (Song et al., 2007), *GL6* (Wang et al., 2019a), and *DEP3* (Qiao et al., 2011) have been previously cloned. On chromosome 7, relatively fewer QTLs were identified in this study, and a QTL for the NFGPP located in the region of *GL7*/*GW7* (Wang et al., 2015a; Wang et al., 2015b) was detected. In summary, the effects of many grain size genes can be measured in this study, and the QTLs detected for panicle-related traits were mostly overlapped with the grain size QTLs previously reported by Ying et al. (2018), which indicated that the pleiotropy of the genes. Zhang et al. (2012b) indicated the pleiotropism of *HD1* allele on HD, plant height, and yield traits in rice. Zhang et al. (2020) found that the regions of several QTLs for grain weight influence HD between the two parental varieties. Xie et al. (2020) also reported the pleiotropic effects of the rice florigen gene *RFT1* on the amino acid content of unmilled rice. Ouyang et al. (2020) indicated that *OsHG3* can affect rice palea development, grain yield, and quality at the same time. It has been proved that many QTLs/genes, especially QTLs for HD, always affect other yield-related traits at the same time, showing the pleiotropic effects of the QTLs. More attention should be paid to the application of the genes/QTLs with pleiotropy effects in further research on the genetic mechanisms and marker-assisted breeding in the future.

More than 20 grain size genes have been cloned till now, and most of them can influence grain yield-related traits, such as *GS5* (Li et al., 2011b) and *GW5* (Weng et al., 2008) on chromosome 5, *GW8*/*OsSPL16* (Wang et al., 2012) at the end of chromosome 8, and *GS9* (Zhao et al., 2018) on chromosome 9. However, no related QTLs were detected in the regions of *GS5*, *GW5*, *GW8*, and *GS9*, and very few QTLs were detected on chromosomes 4, 5, 8, 9, 11, and 12, while no QTLs were detected on chromosome 10. An advanced population should be developed to increase the genetic differences and to improve the sensitivity of QTL detection.

In this study, QTLs detected for panicle-related traits in the RIL population were mostly overlapped, indicating the validity of phenotypic and genotypic data, as well as the accuracy of the detection methods. Almost all the QTLs with a LOD value of more than 5.0 were repeatedly detected, indicating the stability of the effect of the QTLs. Either by PCR-based low-density mapping or by sequencing-genotyping high-density mapping, the highest number of QTLs were detected on chromosome 3, followed by chromosomes 1 and 2. Among the 93 regions where these QTLs were located, only 19 regions contained previously cloned genes for panicle-related traits. Therefore, the majority of the QTLs reported in this study should be further studied to fine-map and clone new genes controlling panicle development, especially the QTLs in the JD1006–JD1007 and RM1148–RM5556 regions. Pyramiding the beneficial QTLs will be very useful for the yield improvement in marker-assisted breeding in rice.

## Conclusions

More than 100 QTLs for panicle-related traits were detected by either PCR-based genetic map or high-density linkage map using the RIL population, indicating PCR-based genetic map and high-density linkage map were both stable and effective for mapping of QTLs for panicle-related traits. Most of the QTLs were repeatedly identified across the two years, indicating the stability of the QTL effect. Several QTL clusters were identified on chromosomes 1, 2, 3, 6, and 7, indicating the pleiotropy of the QTLs. Cloned genes for grain number or grain size have been reported in part of the intervals for these QTLs. Further fine-mapping and cloning of the newly detected QTLs should be conducted which will help improve grain yield in rice.

## Supplemental Information

10.7717/peerj.12504/supp-1 Supplemental Information 1 QTLs detected by high-density mapping in 2015. HD, Heading date; PL, Panicle length; NPB, Number of primary branches; NSB, Number of secondary branches; NGPP, Number of grains per panicle; NPPP, Number of panicles per plant; NFGPP, Number of filled grains per panicle; SSR, Seed-setting rate; GYPP, Grain yield per plant; *A*, Additive effect of replacing a Huannghuazhan allele with a JZ1560 allele; *R*^2^, Proportion of the phenotypic variation explained by the QTL.

10.7717/peerj.12504/supp-2 Supplemental Information 2 QTLs detected by high-density mapping in 2016. HD, Heading date; PL, Panicle length; NPB, Number of primary branches; NSB, Number of secondary branches; NGPP, Number of grains per panicle; NPPP, Number of panicles per plant; NFGPP, Number of filled grains per panicle; SSR, Seed-setting rate; GYPP, Grain yield per plant; *A*, Additive effect of replacing a Huannghuazhan allele with a JZ1560 allele; *R*^2^, Proportion of the phenotypic variation explained by the QTL.

10.7717/peerj.12504/supp-3 Supplemental Information 3 Genotype and phenotype raw data in 2015.

10.7717/peerj.12504/supp-4 Supplemental Information 4 Genotype and phenotype raw data in 2016.

## Additional Information and Declarations

### Data Availability

The following information was supplied regarding data availability: The raw data is available as a Supplemental File.
