# Supplementary material for: Detection of QTLs for panicle-related traits using an indica × japonica recombinant inbred line population in rice
Source: PeerJ. 2021 Nov 29;9:e12504. doi: 10.7717/peerj.12504 (PMC8638570; doi:10.7717/peerj.12504)
Supplement: Supplemental Information 1 — HD, Heading date; PL, Panicle length; NPB, Number of primary branches; NSB, Number of secondary branches; NGPP, Number of grains per panicle; NPPP, Number of panicles per plant; NFGPP, Number of filled grains per panicle; SSR, Seed-setting rate; GYPP, Grain yield per plant; A, Additive effect of replacing a Huannghuazhan allele with a JZ1560 allele; R2, Proportion of the phenotypic variation explained by the QTL. [file peerj-09-12504-s001.docx]

| Table S1. QTLs detected by high-density mapping in 2015. | | | | | | | | | | |
| --- | --- | --- | --- | --- | --- | --- | --- | --- | --- | --- |
| Trait | Chr | Position | | | Locus-start |  | Locus-stop | LOD | *A* | *R*^2^ |
| HD | 1 | 36.46 | – | 36.89 | Marker37137 | – | Marker37507 | 8.375 | 1.695 | 3.338 |
| HD | 2 | 175.25 | – | 176.33 | Marker441370 | – | Marker442155 | 3.305 | -1.836 | 3.917 |
| HD | 3 | 6.70 | – | 7.43 | Marker449893 | – | Marker450508 | 21.573 | 4.427 | 22.782 |
| HD | 3 | 57.82 | – | 58.62 | Marker497839 | – | Marker500332 | 2.768 | -1.060 | 1.307 |
| HD | 3 | 111.16 | – | 136.66 | Marker599971 | – | Marker625523 | 2.922 | 2.086 | 5.057 |
| HD | 7 | 42.02 | – | 93.25 | Marker1156658 | – | Marker1255919 | 3.006 | 2.322 | 6.267 |
| HD | 7 | 137.20 | – | 144.80 | Marker1308776 | – | Marker1313127 | 2.647 | 1.217 | 1.721 |
| HD | 8 | 38.33 | – | 38.51 | Marker1330696 | – | Marker1330654 | 35.134 | -5.468 | 34.755 |
| PL | 1 | 255.66 | – | 256.20 | Marker206041 | – | Marker207342 | 6.361 | -1.039 | 9.385 |
| PL | 2 | 55.42 | – | 106.27 | Marker279355 | – | Marker337189 | 4.394 | 0.863 | 6.477 |
| PL | 2 | 147.12 | – | 152.32 | Marker402318 | – | Marker412403 | 3.047 | -0.807 | 5.661 |
| PL | 3 | 152.00 | – | 152.74 | Marker642132 | – | Marker642647 | 3.592 | -0.301 | 0.790 |
| PL | 3 | 167.47 | – | 168.06 | Marker650748 | – | Marker651024 | 7.116 | 0.824 | 5.906 |
| PL | 4 | 100.12 | – | 101.09 | Marker746306 | – | Marker747242 | 2.511 | -0.652 | 3.701 |
| PL | 9 | 132.62 | – | 137.82 | Marker1607633 | – | Marker1608876 | 2.747 | 0.587 | 2.995 |
| NPB | 1 | 29.64 | – | 30.25 | Marker21929 | – | Marker21927 | 9.708 | 0.747 | 13.818 |
| NPB | 2 | 150.89 | – | 153.04 | Marker409697 | – | Marker415060 | 2.722 | -0.397 | 3.913 |
| NPB | 3 | 7.61 | – | 8.36 | Marker450687 | – | Marker451639 | 5.303 | 0.535 | 7.085 |
| NPB | 5 | 173.41 | – | 181.68 | Marker963176 | – | Marker968984 | 2.868 | -0.307 | 2.333 |
| NPB | 6 | 83.34 | – | 84.27 | Marker1095464 | – | Marker1096868 | 2.745 | 0.403 | 4.026 |
| NPB | 9 | 151.15 | – | 152.25 | Marker1614285 | – | Marker1614766 | 2.974 | 0.435 | 4.698 |
| NPB | 11 | 5.86 | – | 6.23 | Marker1745886 | – | Marker1746140 | 3.982 | 0.456 | 5.151 |
| NPB | 12 | 44.01 | – | 52.19 | Marker1951509 | – | Marker1971628 | 3.105 | 0.622 | 9.580 |
| NSB | 1 | 37.91 | – | 37.91 | Marker38094 | – | Marker38094 | 12.080 | 4.730 | 15.985 |
| NSB | 2 | 40.56 | – | 41.10 | Marker269042 | – | Marker269624 | 5.561 | 2.655 | 5.038 |
| NSB | 6 | 57.12 | – | 57.87 | Marker1025471 | – | Marker1028015 | 4.558 | 2.279 | 3.712 |
| NSB | 8 | 132.47 | – | 132.67 | Marker1452359 | – | Marker1452667 | 2.680 | 1.952 | 2.723 |
| NGPP | 1 | 30.25 | – | 30.97 | Marker22645 | – | Marker23887 | 9.957 | 18.467 | 12.282 |
| NGPP | 2 | 96.99 | – | 97.71 | Marker323226 | – | Marker324444 | 4.473 | 9.845 | 3.491 |
| NGPP | 6 | 57.12 | – | 57.87 | Marker1025471 | – | Marker1028015 | 4.461 | 12.534 | 5.658 |
| NPPP | 1 | 21.13 | – | 21.49 | Marker16499 | – | Marker16994 | 4.997 | -0.683 | 6.075 |
| NPPP | 2 | 39.63 | – | 40.13 | Marker264180 | – | Marker264179 | 8.424 | 0.983 | 12.566 |
| NPPP | 6 | 105.60 | – | 106.73 | Marker1118505 | – | Marker1120642 | 2.686 | 0.542 | 3.823 |
| NPPP | 8 | 107.69 | – | 108.43 | Marker1436858 | – | Marker1438234 | 3.646 | -0.639 | 5.311 |
| NFGPP | 1 | 36.46 | – | 36.89 | Marker37137 | – | Marker37507 | 6.012 | 94.150 | 6.910 |
| NFGPP | 2 | 36.32 | – | 40.13 | Marker261585 | – | Marker264179 | 15.637 | 151.613 | 17.919 |
| NFGPP | 3 | 7.61 | – | 8.36 | Marker450687 | – | Marker451639 | 4.253 | 72.800 | 4.132 |
| NFGPP | 3 | 156.10 | – | 162.06 | Marker646848 | – | Marker649891 | 3.278 | 61.959 | 2.993 |
| NFGPP | 4 | 189.69 | – | 195.66 | Marker802121 | – | Marker806892 | 2.902 | 68.915 | 3.702 |
| NFGPP | 6 | 105.60 | – | 105.60 | Marker1118505 | – | Marker1118506 | 2.768 | 59.281 | 2.740 |
| SSR | 3 | 5.76 | – | 5.95 | Marker448696 | – | Marker449157 | 10.996 | 6.235 | 12.356 |
| SSR | 3 | 90.64 | – | 91.37 | Marker564666 | – | Marker567336 | 6.299 | 4.520 | 6.494 |
| SSR | 3 | 157.15 | – | 157.34 | Marker647458 | – | Marker647484 | 3.894 | 3.114 | 3.083 |
| SSR | 8 | 39.43 | – | 39.43 | Marker1331214 | – | Marker1331214 | 2.527 | -3.419 | 3.714 |
| GYPP | 1 | 36.46 | – | 36.46 | Marker37137 | – | Marker37048 | 2.728 | 1.862 | 3.551 |
| GYPP | 1 | 83.68 | – | 89.00 | Marker46519 | – | Marker44558 | 2.673 | 2.113 | 4.571 |
| GYPP | 2 | 36.32 | – | 39.63 | Marker261585 | – | Marker262537 | 4.465 | 2.201 | 4.962 |
| GYPP | 3 | 7.98 | – | 8.73 | Marker451033 | – | Marker451717 | 7.178 | 2.851 | 8.323 |
| GYPP | 4 | 168.13 | – | 175.36 | Marker785805 | – | Marker793410 | 3.134 | 2.269 | 5.271 |
| GYPP | 5 | 154.43 | – | 170.98 | Marker946308 | – | Marker961173 | 3.066 | 1.868 | 3.574 |
| GYPP | 6 | 111.83 | – | 112.56 | Marker1123872 | – | Marker1124442 | 3.266 | 1.763 | 3.182 |
| GYPP | 8 | 35.17 | – | 40.21 | Marker1330050 | – | Marker1330830 | 2.722 | -1.894 | 3.675 |

HD, Heading date; PL, Panicle length; NPB, Number of primary branches; NSB, Number of secondary branches; NGPP, Number of grains per panicle; NPPP, Number of panicles per plant; NFGPP, Number of filled grains per panicle; SSR, Seed-setting rate; GYPP, Grain yield per plant; *A*, Additive effect of replacing a Huannghuazhan allele with a JZ1560 allele; *R*^2^, Proportion of the phenotypic variation explained by the QTL.
