# Supplementary material for: Detection of QTLs for panicle-related traits using an indica × japonica recombinant inbred line population in rice
Source: PeerJ. 2021 Nov 29;9:e12504. doi: 10.7717/peerj.12504 (PMC8638570; doi:10.7717/peerj.12504)
Supplement: Supplemental Information 2 — HD, Heading date; PL, Panicle length; NPB, Number of primary branches; NSB, Number of secondary branches; NGPP, Number of grains per panicle; NPPP, Number of panicles per plant; NFGPP, Number of filled grains per panicle; SSR, Seed-setting rate; GYPP, Grain yield per plant; A, Additive effect of replacing a Huannghuazhan allele with a JZ1560 allele; R2, Proportion of the phenotypic variation explained by the QTL. [file peerj-09-12504-s002.docx]

| Table S2. QTLs detected by high-density mapping in 2016. | | | | | | | | |
| --- | --- | --- | --- | --- | --- | --- | --- | --- |
| Trait | Chr | Position | Locus-start |  | Locus-stop | LOD | *A* | *R*^2^ |
| HD | 1 | 36.46–36.89 | Marker37048 | – | Marker37507 | 8.136 | 1.652 | 3.625 |
| HD | 3 | 6.70–7.07 | Marker449892 | – | Marker450216 | 13.802 | 3.438 | 15.699 |
| HD | 3 | 112.12–148.93 | Marker601419 | – | Marker639860 | 5.935 | 2.178 | 6.300 |
| HD | 7 | 49.81–89.48 | Marker1170181 | – | Marker1252077 | 4.062 | 2.021 | 5.428 |
| HD | 8 | 38.33–38.51 | Marker1330696 | – | Marker1330654 | 33.683 | -5.153 | 35.278 |
| PL | 1 | 251.30–257.86 | Marker203467 | – | Marker210876 | 3.199 | -0.750 | 4.468 |
| PL | 2 | 92.41–93.09 | Marker321151 | – | Marker321614 | 7.948 | 1.112 | 9.823 |
| PL | 3 | 13.08–13.08 | Marker453435 | – | Marker453430 | 3.695 | 0.734 | 4.277 |
| PL | 3 | 167.47–168.06 | Marker650944 | – | Marker650998 | 2.828 | 0.741 | 4.360 |
| PL | 4 | 1.60–4.68 | Marker652983 | – | Marker654342 | 3.088 | -0.544 | 2.348 |
| PL | 6 | 67.62–68.41 | Marker1039580 | – | Marker1060572 | 4.257 | 0.782 | 4.860 |
| NPB | 1 | 30.61–30.97 | Marker21788 | – | Marker23887 | 5.923 | 0.536 | 6.162 |
| NPB | 2 | 96.46–96.81 | Marker322881 | – | Marker324653 | 9.013 | 0.643 | 8.855 |
| NPB | 2 | 153.04–160.95 | Marker409991 | – | Marker424309 | 2.926 | -0.300 | 1.935 |
| NPB | 3 | 8.36–8.73 | Marker451487 | – | Marker451733 | 4.677 | 0.516 | 5.708 |
| NPB | 5 | 181.14–181.68 | Marker967411 | – | Marker968183 | 2.628 | -0.391 | 3.278 |
| NPB | 6 | 38.00–38.00 | Marker997522 | – | Marker997753 | 3.409 | 0.517 | 5.724 |
| NPB | 9 | 48.01–53.08 | Marker1544953 | – | Marker1550971 | 2.596 | -0.355 | 2.704 |
| NPB | 11 | 6.23–21.19 | Marker1746140 | – | Marker1760926 | 3.217 | 0.520 | 5.784 |
| NPB | 12 | 13.82–15.20 | Marker1923666 | – | Marker1924881 | 3.282 | 0.517 | 5.724 |
| NSB | 1 | 36.46–36.89 | Marker37048 | – | Marker37507 | 16.932 | 5.203 | 17.547 |
| NSB | 1 | 239.62–239.62 | Marker186061 | – | Marker184661 | 2.632 | 2.318 | 3.482 |
| NSB | 2 | 96.46–96.81 | Marker322881 | – | Marker324653 | 12.626 | 3.765 | 9.191 |
| NSB | 3 | 164.09–168.06 | Marker650125 | – | Marker650998 | 7.526 | 3.432 | 7.637 |
| NSB | 6 | 57.68–58.46 | Marker1026956 | – | Marker1047721 | 9.172 | 3.706 | 8.906 |
| NSB | 7 | 134.25–141.20 | Marker1306637 | – | Marker1310253 | 3.217 | -0.599 | 0.232 |
| NGPP | 1 | 36.46–36.89 | Marker37048 | – | Marker37507 | 15.801 | 20.183 | 15.833 |
| NGPP | 2 | 96.46–96.81 | Marker322881 | – | Marker324653 | 11.931 | 16.564 | 10.665 |
| NGPP | 3 | 167.47–167.87 | Marker650944 | – | Marker651334 | 6.562 | 12.953 | 6.521 |
| NGPP | 6 | 51.08–94.26 | Marker1013169 | – | Marker1109064 | 5.853 | 12.468 | 6.042 |
| NPPP | 1 | 29.28–29.64 | Marker20132 | – | Marker21929 | 5.619 | -0.872 | 6.460 |
| NPPP | 2 | 45.74–46.10 | Marker271084 | – | Marker272133 | 9.394 | 0.967 | 7.940 |
| NPPP | 2 | 103.50–104.11 | Marker328970 | – | Marker333155 | 3.693 | -0.004 | 0.000 |
| NPPP | 3 | 0.00–5.76 | Marker444789 | – | Marker449058 | 3.471 | -0.835 | 5.928 |
| NPPP | 6 | 108.09–108.66 | Marker1121715 | – | Marker1121710 | 3.037 | 0.591 | 2.964 |
| NPPP | 8 | 36.28–39.06 | Marker1330552 | – | Marker1330714 | 2.806 | 0.608 | 3.142 |
| NFGPP | 1 | 38.74 | Marker37508 | – | Marker37508 | 10.902 | 117.782 | 12.309 |
| NFGPP | 2 | 40.74–41.46 | Marker267225 | – | Marker270018 | 11.715 | 124.759 | 13.810 |
| NFGPP | 3 | 4.92–48.64 | Marker448514 | – | Marker479366 | 4.963 | 87.604 | 6.809 |
| NFGPP | 3 | 84.13–112.33 | Marker533726 | – | Marker602568 | 3.719 | 82.276 | 6.006 |
| NFGPP | 3 | 161.33–161.80 | Marker649280 | – | Marker649626 | 6.342 | 91.385 | 7.410 |
| NFGPP | 6 | 110.72–110.72 | Marker1123143 | – | Marker1123146 | 2.537 | 44.168 | 1.731 |
| NFGPP | 7 | 105.63–116.28 | Marker1274047 | – | Marker1286195 | 3.556 | 81.154 | 5.843 |
| SSR | 1 | 50.18–50.89 | Marker38706 | – | Marker38705 | 6.011 | 7.272 | 10.573 |
| SSR | 2 | 37.36–105.01 | Marker261592 | – | Marker336271 | 4.488 | 5.472 | 5.986 |
| SSR | 3 | 14.54–15.01 | Marker455664 | – | Marker453950 | 6.170 | 6.274 | 7.870 |
| SSR | 3 | 123.82–124.36 | Marker613845 | – | Marker614445 | 6.225 | 6.319 | 7.982 |
| SSR | 4 | 164.11–169.03 | Marker779713 | – | Marker789266 | 2.608 | 4.319 | 3.729 |
| SSR | 5 | 162.84–170.22 | Marker956807 | – | Marker960514 | 2.987 | 4.428 | 3.920 |
| SSR | 7 | 140.06–144.80 | Marker1309708 | – | Marker1313587 | 4.056 | 5.318 | 5.654 |
| SSR | 9 | 127.28–134.93 | Marker1604838 | – | Marker1608063 | 2.784 | -4.256 | 3.622 |
| GYPP | 1 | 38.74 | Marker37508 | – | Marker37508 | 6.119 | 2.719 | 10.505 |
| GYPP | 2 | 36.57–39.63 | Marker261586 | – | Marker262123 | 5.574 | 2.012 | 5.756 |
| GYPP | 3 | 30.76 | Marker465530 | – | Marker465530 | 5.216 | 2.452 | 8.543 |
| GYPP | 3 | 117.42–125.63 | Marker607329 | – | Marker615204 | 4.107 | 2.134 | 6.474 |
| GYPP | 3 | 154.57–162.06 | Marker645679 | – | Marker649891 | 3.890 | 1.881 | 5.027 |
| GYPP | 4 | 172.65–179.19 | Marker791790 | – | Marker795626 | 2.988 | 1.717 | 4.192 |
| GYPP | 5 | 154.43–173.52 | Marker946309 | – | Marker963334 | 4.472 | 1.739 | 4.301 |
| GYPP | 7 | 100.96–116.28 | Marker1265411 | – | Marker1286195 | 3.521 | 1.897 | 5.113 |
| GYPP | 9 | 134.93–134.93 | Marker1608062 | – | Marker1608063 | 2.766 | -1.452 | 2.997 |

HD, Heading date; PL, Panicle length; NPB, Number of primary branches; NSB, Number of secondary branches; NGPP, Number of grains per panicle; NPPP, Number of panicles per plant; NFGPP, Number of filled grains per panicle; SSR, Seed-setting rate; GYPP, Grain yield per plant; *A*, Additive effect of replacing a Huannghuazhan allele with a JZ1560 allele; *R*^2^, Proportion of the phenotypic variation explained by the QTL.
